# Ligustilide, a major bioactive component of *Angelica sinensis*, promotes bone formation via the GPR30/EGFR pathway

**DOI:** 10.1038/s41598-019-43518-7

**Published:** 2019-05-06

**Authors:** F. Yang, Z. W. Lin, T. Y. Huang, T. T. Chen, J. Cui, M. Y. Li, Y. Q. Hua

**Affiliations:** 1 Jiangsu Key Laboratory for Pharmacology and Safety Evaluation of Chinese Materia Medica, School of Pharmacy, Nanjing University of Chinese Medicine, Nanjing, 210023 China; 2 Jiangsu Collaborative Innovation Center of Chinese Medicinal Resources Industrialization, Nanjing University of Chinese Medicine, Nanjing, 210023 China; 3 School of Pharmacy, Nanjing University of Chinese Medicine, Nanjing, 210023 Jiangsu Province China

**Keywords:** Metabolic bone disease, Health care, Metabolic bone disease, Health care

## Abstract

*Angelica sinensis* (*Oliv*.) *Diels* is a widely-used traditional Chinese herbal medicine in treating osteoporosis. Ligustilide (LIG) is the main component of *A*. *sinensis* and is considered to be the most effective biologically active ingredient in this plant. LIG has been found to have multiple pharmacological activities, such as anti-atherosclerosis, neuroprotection, anticancer, anti-inflammatory and analgesic. However, little is known regarding its anti-osteoporotic effects. The aims of this study were to investigate any protective effect of LIG on bone formation. The results showed that LIG significantly ameliorated inhibition of bone formation in zebrafish caused by prednisolone. LIG promoted osteoblast differentiation, including that of the pre-osteoblastic cell line MC3T3-E1 and bone marrow mesenchymal stem cells. LIG greatly improved the viability of MC3T3-E1 cells exposed to H_2_O_2_, attenuated H_2_O_2_-induced apoptosis and increased the expression of Bcl-2. Furthermore, LIG treatment lead to marked activation of phosphorylated EGFR and ERK1/2. These effects could be obviously inhibited by blocking GPR30 signaling with the specific inhibitor G15. Collectively, the results reveal that GPR30 is a positive switch for LIG to increase bone formation via regulation of EGFR, and these results provide evidence for the potential of LIG to treat osteoporosis.

## Introduction

Osteoporosis (OP) is a systemic bone disease characterized by decreased bone mass and degeneration of bone microstructure, resulting in increased fragility of bone, making it prone to fracture^1,2^. Bone mass regulation depends on the dynamic balance between bone formation and bone resorption, which is affected by the activation of osteoblasts or osteoclasts, respectively^3,4^. The main factors affecting bone homeostasis include sex hormones, glucocorticoid administration, cellular inflammatory factors, mechanical forces, and oxidative stress (OS)^5,6^. A decline in estrogen levels has been considered the crucial mechanism of OP. Recent studies indicated that aging and elevated reactive oxygen species (ROS) level are the primary culprits. ROS significantly affect the formation and survival of osteoclasts (OCs), osteoblasts (OBs), and osteocytes^7,8^. Therefore, reducing the OS-induced OB damage is an effective way to prevent or delay the bone loss in OP.

At present, a series of anti-resorptive or anabolic options are commonly used to prevent osteoporotic fractures, especially bisphosphonates, which are the first choice for clinical treatment^9^. However, long-term use of bisphosphonate and denosumab lead to the risk of side effects, such as atypical subtrochanteric fracture or osteonecrosis of the jaw^10^. Therefore, identification of new drugs to treat OP is a worldwide problem and a hot topic. Traditional Chinese medicine (TCM) has unique advantages in the treatment or prevention of OP, and consequently has attracted widespread attention from scholars all around the world^11^.

*Angelica sinensis* (*Oliv*.) *Diels* has a long history of use in traditional herbal medicine. It is traditionally used for treating female disorders including irregular menstruation, amenorrhea and OP^12^. *A*. *sinensis* is one of the most commonly-used TCMs for the treatment of OP, ranked third and first in the commonly-used TCMs for the treatment of OP and osteoarthritis^13^. Studies confirmed that rats treated with *A*. *sinensis* showed less trabecular bone loss and thicker cortical areas^14^. The volatile oil is one of the main effective components of *A*. *sinensis*, and ligustilide (LIG) is the most abundant component in the volatile oil, accounting for approximately 60%^15^. LIG has been found to have multiple pharmacological activities such as anti-atherosclerosis, neuroprotection, anti-cancer, inhibition of cardiac hypertrophy, anti-inflammation and analgesia^16^. Moreover, LIG has been reported to protect skeletal muscle cells, nerve cells, and endothelial cells from all sorts of damage and to have anti-osteoarthritis effects^17–20^. However, it is unclear whether the therapeutic effect of *A*. *sinensis* on OP is related to LIG. This study found that LIG promotes bone formation by regulating the GPR30/EGFR signaling pathway. Our results provide new insights into the therapeutic mechanism of *A*. *sinensis* in OP, and provide a basis for its application in the treatment of OP.

## Results

### LIG improved bone mass in a zebrafish bone-formation-inhibiting model

**Figure 1:**
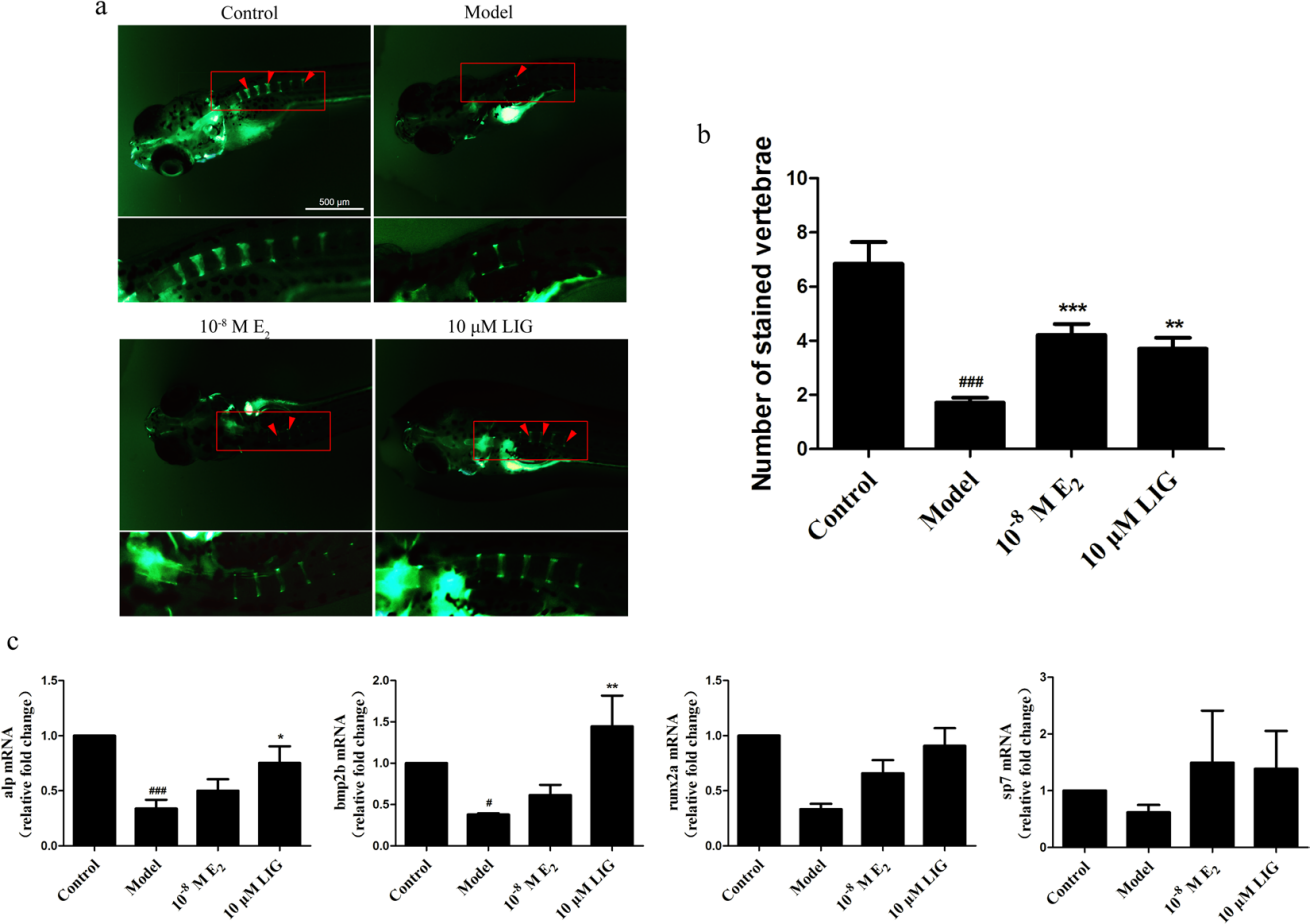
LIG increases bone mass *in vivo*. (**a**) Calcein staining of zebrafish larvae at 9 dpf. Scale bars, 500 μm. Arrowheads, bone calcein staining. (**b**) Number of calcein-stained vertebrae in zebrafish at 9 dpf. Error bars indicate s.e.m., n = 13 for each group. ^###^*P* ≤ 0.001 vs. control group. **P ≤ 0.01, ***P ≤ 0.001 vs. model group. (**c**) The expression mRNA for Alp, Runx2a, sp7, and BMP2b were measured by quantitative RT-PCR in zebrafish at 9 dpf. Error bars indicate s.e.m. (n = 4). ^#^*P* ≤ 0.05, ^###^*P* ≤ 0.001 vs. control group. *P ≤ 0.05, **P ≤ 0.01 vs. model group.

A bone-formation-inhibiting model was established in zebrafish by treating with prednisolone (Pred; 25 μM)^21^. There is no obvious malformations in embryonic zebrafish treated with prednisolone for 9 days (Supplementary Figure 1a). LIG at 10 μM concentration has no obvious effect on bone mass in normal zebrafish (Supplementary Figure 1b). We then examined the function of LIG in this model, with 17β-estradiol (E_2_; 10^−8^ M) used as a positive control^22,23^. Compared with the control group, zebrafish at 3 days post-fertilization (3 dpf) treated with Pred for 6 days showed less bone mass. Compared with the model group, LIG increased the the number of vertebrae and the expression of OB-marker genes (alp, runx2a, sp7 and bmp2b) in zebrafish (*P* < 0.05, *P* < 0.01, *P* < 0.001), showing an equivalent effect to E_2_ (Fig. 1).

### LIG enhanced the osteogenic differentiation of the pre-osteoblastic cell line MC3T3-E1 and bone marrow mesenchymal stem cells (BMSCs)

**Figure 2:**
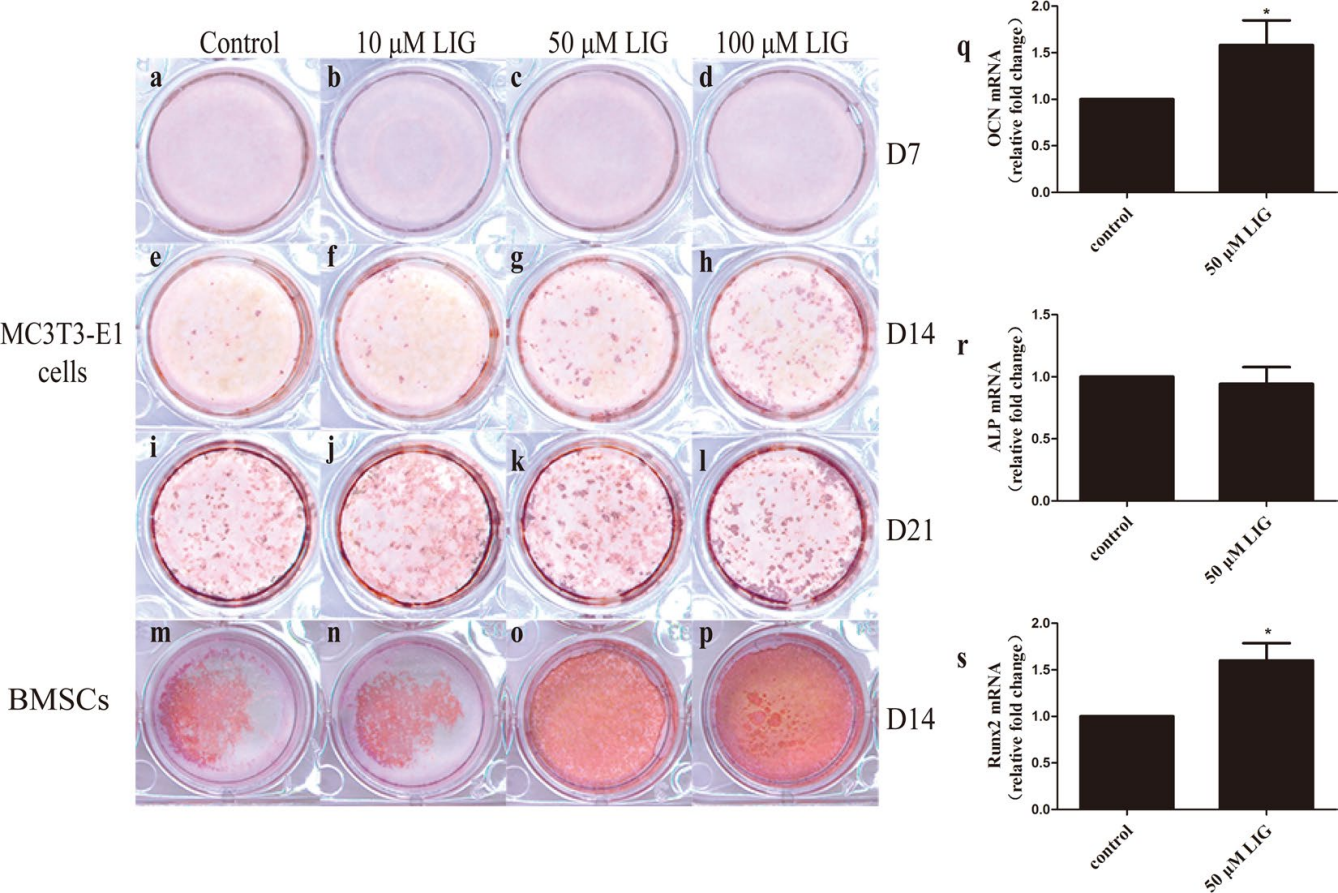
LIG enhances OB differentiation. (**a–l**) Alizarin red staining of MC3T3-E1 cells after 7, 14 and 21 days (D7, 14, 21) in culture under OIM with LIG over the entire culture period. (**m–p**) Alizarin red staining of BMSCs after 14 days (D14) in culture under OIM with LIG over the entire culture period. Pictures taken from a 12-well plate. (**q–s**) The expression mRNA for Alp, Runx2 and OCN were measured by quantitative RT-PCR in BMSCs on the 6th day. Error bars indicate s.e.m. (n = 3). *P ≤ 0.05 vs. control group.

MC3T3-E1 cells and BMSCs were used to explore the effect of LIG on bone formation *in vitro*. Cells were incubated with OB induction medium (OIM)^24^ to induce differentiation. MC3T3-E1 cells displayed a time-dependent development of OB characteristics analogous to *in vivo* bone formation^25^. In these cell cultures, at day 7, no obvious calcified nodules were observed in any group, as assessed by alizarin red staining (Fig. 2a–d). However, by day 14, LIG had significantly enhanced calcified nodule formation compared with the control group (Fig. 2e–h). After 21 days, a large number of calcified nodules had appeared in each group, and there was no obvious difference between the LIG group and the control group (Fig. 2i–l). BMSCs have the ability to differentiate into adipocytes, chondrocytes, and OBs^26,27^, and play a key role in regulating bone homeostasis^28^. On the 14th day, in OIM-induced osteogenic differentiated BMSCs, more calcified nodules were observed. Compared with MC3T3-E1 cells, BMSCs possessed greater osteogenic differentiation ability under the same differentiation conditions (Fig. 2 l,m). LIG also promoted osteogenic differentiation of BMSCs (Fig. 2m–p), which was consistent with the effects of LIG *in vivo*. Compared with the control group, 50 μM LIG significantly promoted the expression of OB-marker genes (Runx2 and OCN) in BMSCs on the 6th day. These results demonstrated that the effect of LIG on improving bone mass might be achieved by increasing OB differentiation.

### LIG attenuated H_2_O_2_-induced apoptosis in OBs

**Figure 3:**
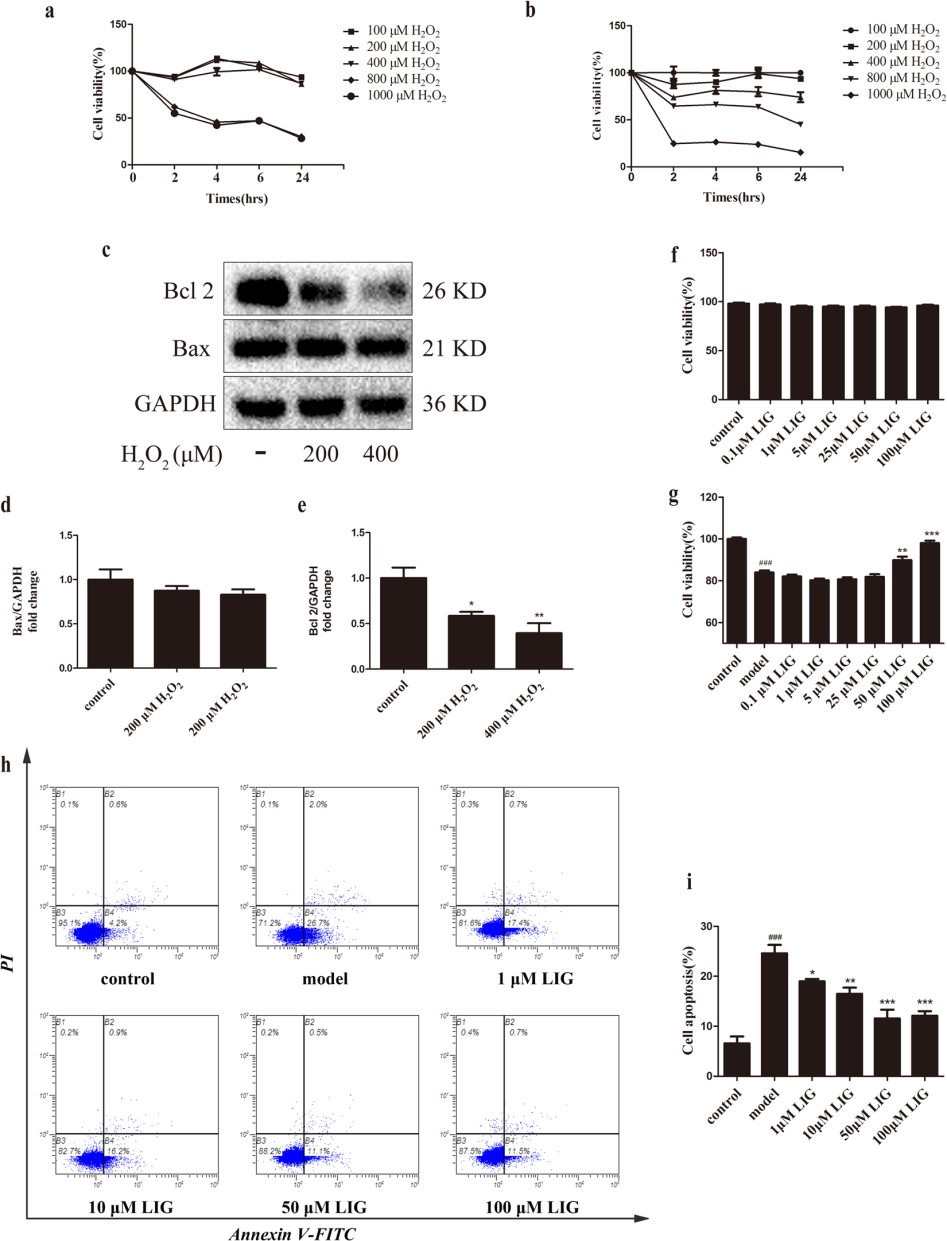
LIG attenuates H_2_O_2_-induced apoptosis in MC3T3-E1 cells. (**a**,**b**) Cell viability was determined by MTT reduction in MC3T3-E1 cells in the presence of H_2_O_2_. Error bars indicate s.e.m. (n = 6). (**c**) Representative immunoreactive bands for Bcl-2 and Bax in MC3T3-E1 cells in the presence of H_2_O_2_. Quantification of immunoreactive bands for Bcl-2 (**d**) and Bax (**e**) relative to GAPDH. Error bars indicate s.e.m. (n = 3). (**f**) and (**g**) Cell viability was determined by MTT reduction in MC3T3-E1 cells with LIG in the presence or absence of H_2_O_2_. Error bars indicate s.e.m. (n = 18). (**h**) and (**i**) Flow cytometric quantification of apoptosis. Error bars indicate s.e.m. (n = 3). ^###^*P* ≤ 0.001 vs. control group. *P ≤ 0.05, **P ≤ 0.01, ***P ≤ 0.001 vs. model group.

A model of peroxidation damage to MC3T3-E1 cells^29^ was established to determine the protective effect of LIG on pre-osteoblasts. There was no obvious effect on cell viability after treating with no more than 400 μM H_2_O_2_ for 4 hours. After that, the cells were cultured for 24 h with complete medium without H_2_O_2_, and the cell survival rate decreased in a concentration-dependent manner (Fig. 3a,b). Compared with the untreated control group, the cell viability of 200 μM and 400 μM H_2_O_2_-treated groups significantly decreased approximately 30 to 40%. The cell survival rate of 800 μM and 1,000 μM H_2_O_2_-treated groups was less than 20%. These results suggested that H_2_O_2_ damage to cells was a continuous process, and that damage continued to occur even when the level of oxidation in the external environment was reduced.

The level of the anti-apoptotic protein Bcl-2 in H_2_O_2_-induced OB apoptosis was measured (Fig. 3c–e). Compared with the untreated control group, 200 μM and 400 μM H_2_O_2_ both significantly decreased the Bcl-2 expression (*P* < 0.05), while no remarkable effect was observed in the expression of the pro-apoptotic protein Bax (Fig. 3d,e).

Although LIG is known to reduce apoptosis in a variety of non-cancerous cells, its effects on oxidative stress-induced OB apoptosis have not been elucidated. LIG at concentrations below 100 μM for 24 h had no effect on MC3T3-E1 cell viability (Fig. 3f). However, when MC3T3-E1 cells were treated with LIG after H_2_O_2_ exposure and cell viability was measured by MTT assay, the results showed that 50 or 100 μM LIG improved the cell survival rate (*P* < 0.01) (Fig. 3g). Apoptosis was evaluated by flow cytometry, which showed that LIG markedly inhibited apoptosis at concentrations from 1 μM to 100 μM (*P* < 0.05, *P* < 0.01, *P* < 0.001) (Fig. 3h,i).

### LIG attenuated H_2_O_2_-induced apoptosis and promoted differentiation in OBs through GPR30

**Figure 4:**
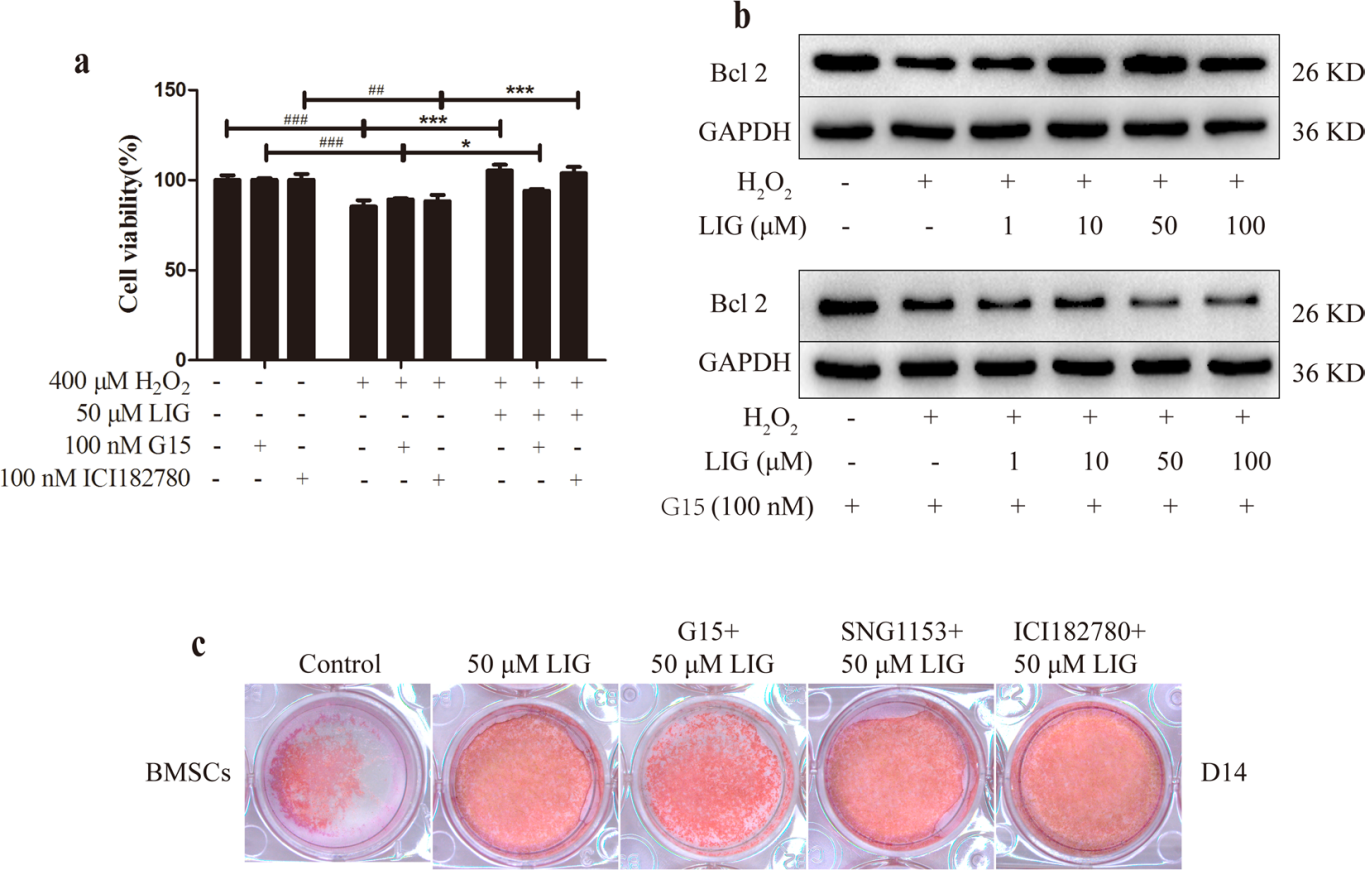
LIG attenuates H_2_O_2_-induced apoptosis and promotes differentiation in OBs through GPR30. (**a**) Cell viability was measured by MTT reduction in OBs with LIG, G15 and ICI182780 in the presence of H_2_O_2_. Error bars indicate s.e.m. (n = 6); ^##^*P* ≤ 0.01, ^###^*P* ≤ 0.001 vs. control group. *P ≤ 0.05, **P ≤ 0.01, ***P ≤ 0.001 vs. model group. (**b**) Representative immunoblotting images for Bcl-2 in MC3T3-E1 cells with LIG and G15 under oxidative stress. (**c**) Alizarin red staining of BMSCs after 14 days (D14) in culture under OIM with LIG and co-treated with 100 nM G15, 100 nM ICI182780 or 100 nM SNG1153 over the entire culture period.

To understand the mechanism of the anti-apoptotic effect of LIG in MC3T3-E1 cells, the effects of LIG, G15 and ICI182780 on H_2_O_2_-induced damage were observed by MTT and immunoblotting. When co-treated with 100 nM G15, the GPR30 antagonist, the promotional effect of LIG on cell survival was obviously restrained. This blocking effect was not observed in the 100 nM ICI182780-treated group (Fig. 4a). Previous studies have pointed out that G1, a GPR30 agonist, decreased ROS levels in endothelial cells^30^. GPR30 might thus have a protective effect in OBs under oxidative stress. LIG significantly increased the expression of Bcl-2 in MC3T3-E1 cells after peroxidation injury, and this effect was completely abolished by co-treatment with G15 (Fig. 4b).

To explore the mechanism underlying the promotion of osteogenic differentiation by LIG, BMSCs were selected which were more susceptible to differentiation. Compared with the control group, the number of calcified nodules was significantly increased when the BMSCs were treated with 50 μM LIG alone or combined with 100 nM SNG1153 and 100 nM ICI182780. In contrast, compared with the LIG treatment group, the number of calcified nodules was significantly reduced when the BMSCs were treated with LIG combined with 100 nM G15 (Fig. 4c). These results indicated that the promotional effect of LIG on BMSC differentiation was mediated through GPR30.

### The anti-apoptotic effect of LIG on OBs was associated with the GPR30/EGFR pathway

**Figure 5:**
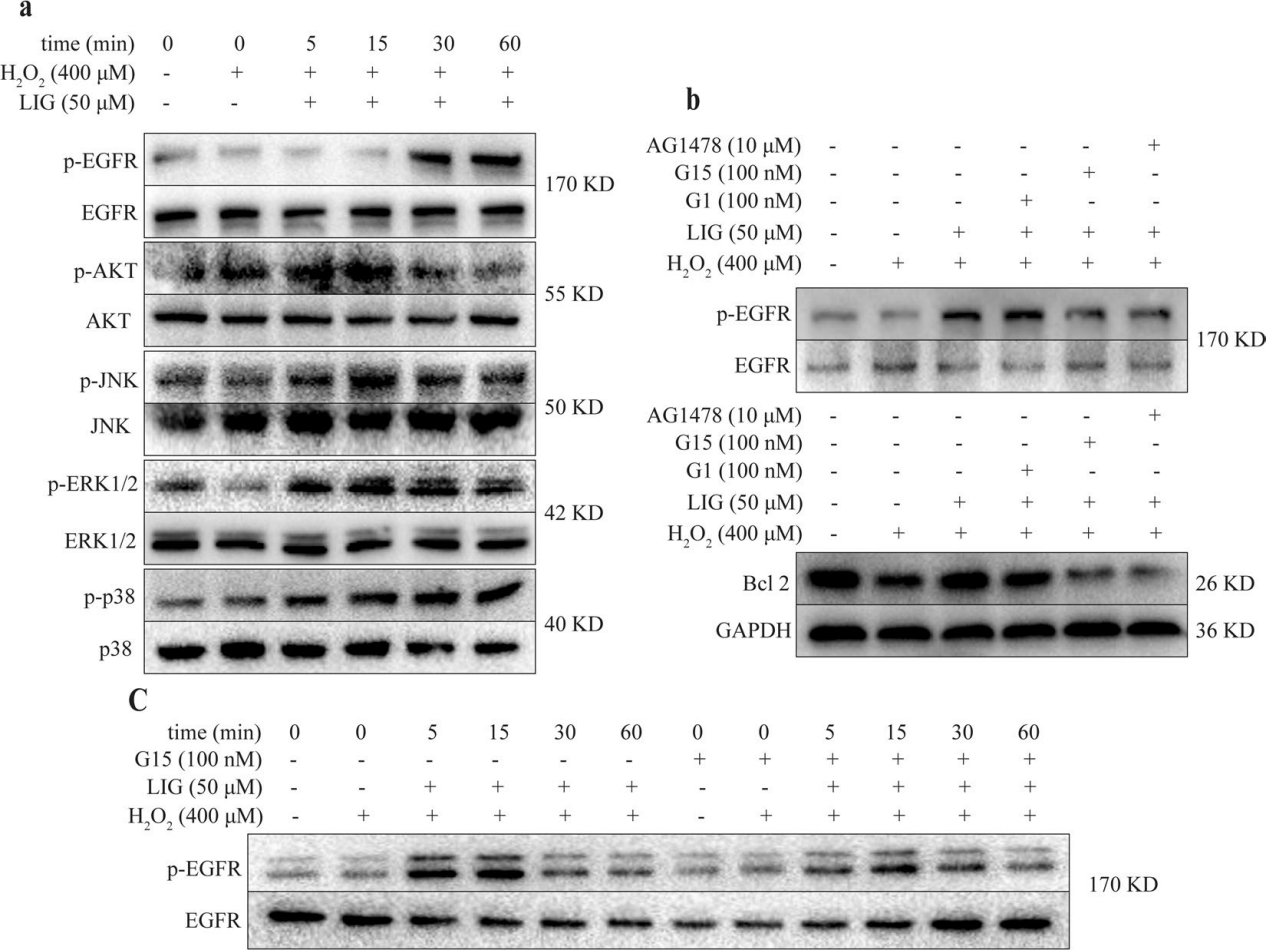
Involvement of GPR30/EGFR/ERK signaling in LIG inhibition of H_2_O_2_-induced apoptosis in MC3T3-E1 cells. (**a**) Time course of LIG effects on the levels of Akt, p38, JNK, ERK1/2 and EGFR in MC3T3-E1 cells. (**b**) LIG and G1 (100 nM) increased the levels of phosphorylated EGFR (p-EGFR) in MC3T3-E1 cells at 30 min. G15 (100 nM) abolished the effects of LIG and G1 (100 nM). (**c**) LIG increased the levels of phosphorylated ERK1/2 (p-ERK1/2) in MC3T3-E1 cells. G15 abolished the effects of LIG. (**d**) G15 (100 nM) and AG1478 (10 μM) inhibited the level of Bcl-2 induced by LIG in MC3T3-E1 cells.

As a G protein-coupled estrogen receptor, GPR30 mediates non-genomic rapid responses through activation of the EGFR, MAPK (ERK/p38/JNK), and PI3K/Akt signaling pathways^31,32^. In addition, GPR30 also affects cell proliferation and differentiation by activating EGFR^33^. Within 1 h, LIG (50 μM) significantly promoted activation of the PI3K/Akt and MAPK pathways and phosphorylation of EGFR in MC3T3-E1 cells under oxidative stress (Fig. 5a). It is known that activation of the GPR30/EGFR/ERK pathway promotes cell proliferation and inhibits apoptosis. Treatment with 50 μM LIG and 100 nM G1, the GPR30 agonist, markedly increased the levels of p-EGFR in OBs (Fig. 5b). Co-treatment of 50 μM LIG and 100 nM G15 attenuated activation of the EGFR and ERK1/2 (Fig. 5b,c), while 100 nM G15 and 10 μM AG1478, a specific inhibitor of EGFR^34,35^, inhibited accumulation of the anti-apoptotic protein Bcl-2 compared with the 50 μM LIG group (Fig. 5d). These results suggested that LIG might exert anti-apoptotic effects in OBs through the Akt, MAPK and GPR30/EGFR signaling pathways.

## Discussion

Our study demonstrated a pharmacological role of LIG in regulation of bone formation and bone mass. Particularly, we found that LIG promotes OB differentiation and prevents pre-osteoblast apoptosis through the GPR30/EGFR pathway.

Estrogen plays a critical role in regulating bone growth through the estrogen receptor^36^. A considerable body of data suggests that the mechanism of estrogen action involves binding to two types of nuclear estrogen receptors (ERα and ERβ)^37^. Recent findings suggested that the novel membrane estrogen receptor GPR30 is involved in non-genomic estrogen signaling^38,39^. GPR30 has been reported to be an essential membrane estrogen receptor for bone development participating in proliferation and differentiation in OBs. G1, a specific agonist of GPR30, promotes bone formation, and G15, a specific antagonist of GPR30, antagonizes the positive effect of G1 on bone^31^. Studies have confirmed that GPR30 is necessary for estradiol or raloxifene-induced proliferation of the human fetal osteoblast hFOB cells^40^. The latest research showed that treatment with 17β-estradiol resulted in the expression of GPR30 and enhanced mitophagy through the GPR30 and ERK1/2 signaling pathways in OBs^41^. In this study, LIG at osteogenic concentrations was unable to activate nuclear ERs including ERα and ERβ, but obviously stimulated GPR30. Among the compounds identified, LIG is thought to be one of the most biologically-active components of *A*. *sinensis*^42^. This is the first demonstration of selective activation of GPR30 in OBs by a monomeric ingredient of *A*. *sinensis*.

Signaling mechanisms of GPR30 regulate transcriptional activity involving cAMP, ERK and PI3K^32^. In addition, rapid signaling through GPR30 occurred via transactivation of the epidermal growth factor receptor (EGFR) and involved non-receptor tyrosine kinases of the Src family^33,43^. EGFR activation leads to multiple downstream events, including the activation of PLC, MAPKs, and PI3Ks^33^. This experiment demonstrated that LIG significantly promoted activation of the EGFR, Akt, and MAPK pathways, downstream of GPR30, under oxidative stress in MC3T3-E1 cells. EGFR signaling significantly increased the number of OBs by modulating the proliferation and apoptosis of osteogenic progenitor cells to stimulate bone formation^44^. Studies have found that EGFR controlled bone development by negatively regulating mTOR signaling during the process of OB differentiation^45^. EGF-induced ERK activation in MC3T3-E1 OBs ultimately leads to proliferation and differentiation^46^. Therefore, the mechanism of LIG might be exerted though complex regulation of OBs survival and apoptosis under oxidative stress via the GPR30/EGFR pathway.

*A*. *sinensis* is one of the most important TCMs commonly used to invigorate the circulation of blood. With increasing age and associated damage to lipid peroxidation, there would inevitably be microcirculation disturbance and abnormal hemorheology. LIG might be one of the key active ingredients of *A*. *sinensis* to act on blood circulation. LIG is known to improve cellular antioxidant defense and inhibit H_2_O_2_-induced apoptosis in PC12 cells^47^. OS-induced OBs apoptosis is crucial to the development of OP^48^. In this study, LIG significantly increased the activity of OBs damaged by H_2_O_2_ and decreased the apoptosis rate, which might lead to a beneficial effect on bone formation. The antioxidant damage of LIG mentioned above might be related to the blood-activating effect of *A*. *sinensis*.

The zebrafish model was employed in this study to investigate the bone formation effects of LIG *in vivo*. Because of the molecular and cellular conservation of skeletal development, zebrafish are considered to be an attractive model for *in vivo* screening studies when studying human diseases^49,50^. Low-dose dexamethasone (10 nM) increases ALP content in OBs and promotes OB differentiation^24,51^. In contrast, high-dose glucocorticoids such as prednisolone (25 μM) have been found to have inhibitory effects on bone formation in zebrafish. During ontogenetic growth^52,53^, homeostasis, and regeneration of zebrafish bone^21^, prednisolone affect the amount, activity and differentiation of OBs, OCs, and immune cells. Here, we showed a therapeutic role of LIG in prednisolone-induced inhibition of bone formation in zebrafish, which was consistent with the effects of LIG *in vitro*.

This study has shown that LIG increases bone mass by promoting OB differentiation and anti-peroxidative damage, and its mechanism might be related to GPR30. The above data indicate that LIG is a beneficial agent in preventing OP by enhancing bone formation. This suggests that it might be one of the active components of *A*. *sinensis* functioning in clinical treatment of diseases such as OP and osteoarthritis, and provides more evidence for the clinical application of *A*. *sinensis* to treat this type of disease. Thus, understanding of the biochemical mechanisms in the GPR30 pathway triggered by LIG might provide some clue for interpreting the action of TCM on bone formation, and might offer more strategies and targets for the treatment of OP.

## Methods

### Zebrafish studies

Zebrafish (*Danio rerio*) (Ezerinka, NanJing, China) were housed at 28 °C, in a 14 h light and 10 h dark cycle. Zebrafish embryos were collected following natural spawning and raised at 28 °C in E3^24^ solution (5 mM NaCl, 0.17 mM KCl, 0.33 mM CaCl, 0.33 mM MgSO_4_) according to standard protocols. All procedures involving animals were approved by the Animal Care and Use Committee of Nanjing University of Chinese Medicine and strictly performed according to the Guide for the Care and Use of Laboratory Animals.

### Prednisolone-induced inhibition of bone formation in zebrafish

Pred (Yuanye, Shanghai, China) was dissolved at 5 mM in 20% dimethyl sulfoxide (DMSO) to create a stock solution. Zebrafish larvae were treated with 25 μM Pred or a corresponding amount of DMSO as control in E3 solution for 6 days, starting at 3 dpf; 10^−8^ M E_2_ (Sigma) was used as the positive control. To evaluate bone mass in larvae, live zebrafish were incubated for 1 h in 0.2% calcein (Yuanye) in distilled water (dH_2_O) (pH 7), then rinsed in water three times, and incubated in dH_2_O for 1 h. The zebrafish were anesthetized with 0.02% Tricaine (MS222) and imaged under a fluorescence microscope (Leica, M205 FA, Leica Microsystems Ltd., Wetzlar Germany). Measurements of intracranial bone area were carried out using the Plot Profile tool in Image J^21^.

### Cell culture

The pre-osteoblasts (MC3T3-E1 cells) used in this study were obtained from the cell library of Shanghai Chinese Academy of Sciences (Shanghai, China). Bone marrow mesenchymal stem cells (BMSCs) were isolated from the femurs of neonatal rats (1 to 2 weeks old) following the method described by Huang^54^, with slight modifications. BMSCs were cultured in α-MEM (Gibco, Grand Island, NY, USA). MC3T3-E1 cells and BMSCs were maintained in α-MEM supplemented with 10% fetal bovine serum (FBS; Gibco) and 1% penicillin–streptomycin (P/S; TransGen Biotech, Beijing, China) in a humidified incubator at 37 °C with 5% CO_2_.

### OB differentiation

For OB differentiation, MC3T3-E1 cells and BMSCs were seeded into 12-well plates (1 × 10^5^ cells/well) and induced with OB induction medium (OIM)^24^ consisting of α-MEM containing 10 mM β-glycerophosphate (Sigma, St. Louis, MO, USA), 10 nM dexamethasone (Dex; Sigma), and 50 mg/mL L-ascorbic acid (Sigma) supplemented with 10% FBS and 1% P/S. Mineralized matrix formation by OB was observed by alizarin red staining (Servicebio, Wuhan, China) examined under a stereomicroscope (ZEISS, Stemi 2000-C; Carl Zeiss Microscopy GmbH, Jena, Germany).

### Cell treatments

The final concentrations of the compounds were as follows: H_2_O_2_ (100, 200, 400, 800, 1,000 and 2,000 μM; Ningshi, Nanjing, China), the GPR30 antagonist G15 (100 nM; Caymanchem, Wuhan, China), the GPR30 agonist G1 (100 nM; Caymanchem), the ER-α 36 antagonist SNG1153 (100 nM; MCE, Princeton, NJ, USA), the ER antagonist ICI182780 (100 nM; Tocris, Massachusetts, USA), EGFR inhibitor AG1478 (10 μM; Selleckchem, Shanghai, China), and LIG (1, 10, 50, and 100 μM; ZZBio Co. Ltd., Shanghai, China). The final concentration of the vehicle, DMSO, was less than 0.5% in all experiments. MC3T3-E1 cells were treated with or without H_2_O_2_ and the indicated test compounds for various times, according to the experimental protocol.

### Cell viability assay

MC3T3-E1 cells were seeded into 96-well plates (1 × 10^4^ cells/well) and cultured under various conditions, as indicated for each experiment. Serum-free α-MEM (200 µL) supplemented with 20 µL MTT solution (5 mg/mL; NeoFroxx, Einhausen, Germany) was added to each well, and cells were further incubated at 37 °C. After 4 h, the MTT medium was removed, and the formazan crystals were dissolved in 150 µL DMSO. The absorbance of the plates was then measured in a microplate reader (BioTek, SyneRgy2; BioTek, Winooski, VT, USA) at 490 nm.

### Flow cytometry

After treatments, MC3T3-E1 cells were analyzed by Annexin V-FITC/PI double staining, and then cytofluorometric analysis was performed by Flow Cytometry (Beckman Coulter, Brea, CA, USA).

### Western blot analyses

After treatments, MC3T3-E1 cells were lysed in cell lysis buffer (Applygen, Beijing, China). Protein concentration was assayed using the BCA protein Assay kit (Beyotime, Shanghai, China). Aliquots containing 20 μg cell protein were separated on 10% SDS-PAGE gels and transferred to polyvinylidene difluoride membranes (Millipore, Darmstadt, Germany) using the Trans-Blot Turbo system (BioRad, Hercules, CA, USA). Anti-phospho-Akt Ser473 (1:1,000; Proteintech, Chicago, IL, USA), anti-Akt (1:1,000; Proteintech), anti-phospho-EGFR (1:1,000; Cell Signaling Technology, Danvers, MA, USA), anti-EGFR (1:1,000; Abcam, Cambridge, UK), anti-phospho-JNK (1:1,000; Abcam), anti-JNK (1:1,000; Cell Signaling Technology), anti-phospho-ERK1/2 (1:1,000; Affbiotech, Changzhou, China), anti-ERK1/2 (1:1,000; Proteintech), anti-phospho-p38 (1:1,000; Abcam), anti-p38 (1:1,000; Abcam), anti-Bcl-2 (1:1,000; Abcam), anti-Bax (1:1,000; Proteintech) and anti-GAPDH antibodies were used as primary antibodies, followed by addition of an appropriate secondary antibody (1:10,000). After antibody incubation, an enhanced chemiluminescence substrate (Millipore) was added and the protein bands were detected using a Bio-Rad ChemiDoc XRS^+^ Image station (BioRad). The images were quantitated using Image Lab software.

### RT-qPCR

Total RNA was isolated and purified by RNA isolater® Total RNA Extraction Reagent (Vazyme Biotech, Nanjing, China). RNA concentrations were determined using Synergy2 Multi-mode Microplate Reader. cDNA was synthesized from 1 mg total RNA using HiScript® II Q RT SuperMix for qPCR (+g DNA wiper) (Vazyme Biotech, Nanjing, China). Real-time PCR was then conducted with the ABI 7500 Sequencing Detection System (Applied Biosystems, Foster City, CA, United States) and the AceQ® qPCR SYBR® Green Master Mix (Vazyme Biotech, Q111-02). Samples were analyzed using a 7500 fast real time PCR System (Applied Biosystems, Foster City, CA, USA). Two microlitres of each cDNA were subjected to PCR amplification using specific primers as follows: bmp2b (F) 5′-CGGTCAACTCCAACATTCCC-3′, (R) 5′-ATTGTTCTCATCGGCAACCG-3′; sp7 (F) 5′-TGGATTGACCCTCACTGGAC-3′, (R) 5′-GATGGTGCTTCCCGGTTTAC-3′; runx2a (F) 5′-GTGGAGATCATAGCGGACCA-3′, (R) 5′-CTCCCAGAGCCACAACCTTA-3′; alp (F) 5′-GGCCTTACATGAAGCTGTGG-3′, (R) 5′-GTTTCCTCGTGGTGTGTAGC-3′; gapdh (F) 5′-TGGTGCTGGTATTGCTCTCA-3′, (R) 5′-ATGGGAGAATGGTCGCGTAT-3′; Runx2 (F) 5′-CATCCTTCCCTCCGAGACCCTAA-3′, (R) 5′-CCCAACATGGCTGCTCCCTTC-3′; OCN (F) 5′-CTCACTCTGCTGGCCCTGAC-3′, (R) 5′-CACCTTACTGCCCTCCTGCTTG-3′; Alp (F) 5′-AGGGTGGGTTTCTCTCTTGG-3′, (R) 5′-CATGATGGTTGCAGGGTCTG-3′; GAPDH (F) 5′-TGAACGGGAAGCTCACTGG-3′, (R) 5′-TCCACCACCCTGTTGCTGTA-3′.

### Data analysis

All data are displayed as means ± standard error (s.e.m.). All statistical analyses were performed on GraphPad Prism 5.0 (GraphPad Software, San Diego, CA, USA). Statistical analysis with multiple comparisons was performed with one-way ANOVA, and differences between treatment groups were considered significant if *P* < 0.05.

## Supplementary information

Supplementary Information

## Data Availability

All data discussed in this report are available upon request.
