# Supplementary material for: Ligustilide, a major bioactive component of Angelica sinensis, promotes bone formation via the GPR30/EGFR pathway
Source: Sci Rep. 2019 May 6;9:6991. doi: 10.1038/s41598-019-43518-7 (PMC6502875; doi:10.1038/s41598-019-43518-7)

Supplementary Information

Ligustilide, a major bioactive component of *Angelica sinensis*, promotes bone formation via the GPR30/EGFR pathway

Yang F[1, 3], Lin ZW[1,3], Huang TY[1, 3], Chen TT[1, 3], Cui J[1, 3], Li MY[1,3], Hua YQ[1, 2, 3]*

[1] Jiangsu Key Laboratory for Pharmacology and Safety Evaluation of Chinese Materia Medica, School of Pharmacy, Nanjing University of Chinese Medicine, Nanjing 210023, China

[2] Jiangsu Collaborative Innovation Center of Chinese Medicinal Resources Industrialization, Nanjing University of Chinese Medicine, Nanjing 210023, China

[3] School of Pharmacy, Nanjing University of Chinese Medicine, Nanjing 210023, Jiangsu Province, China.

*Corresponding author at: School of Pharmacy, Nanjing University of Chinese Medicine, 138 Xianlin Avenue, Qixia District, Nanjing 210023, Jiangsu Province, China.

Tel.: +86 25 85811531

E-mail address: hua_yq@njucm.edu.cn.

Fig S1.

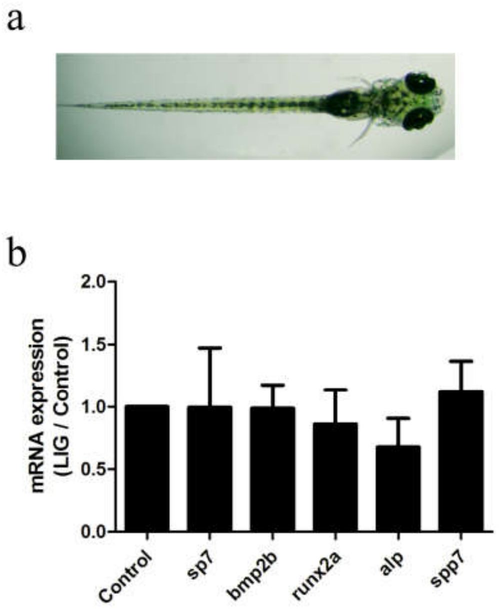

Fig S1. Effects of ligustilide on bone formation in zebrafish. (a) Bright field image of zebrafish at 9 dpf. (b) The mRNA expression of spp1, alp, runx2a, sp7, and bmp2b measured by quantitative RT-PCR in zebrafish at 9 dpf. Error bars indicate S.E.M. (n = 3).

Fig 3.

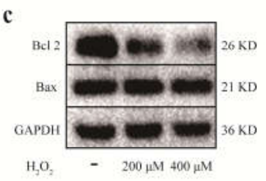

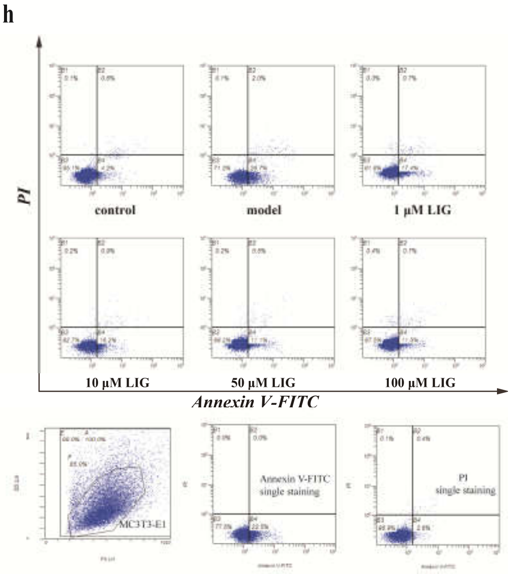

Original films

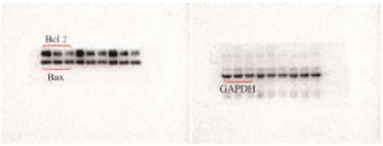

Fig 4.

b

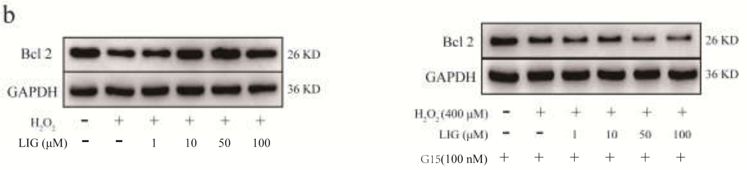

Original films

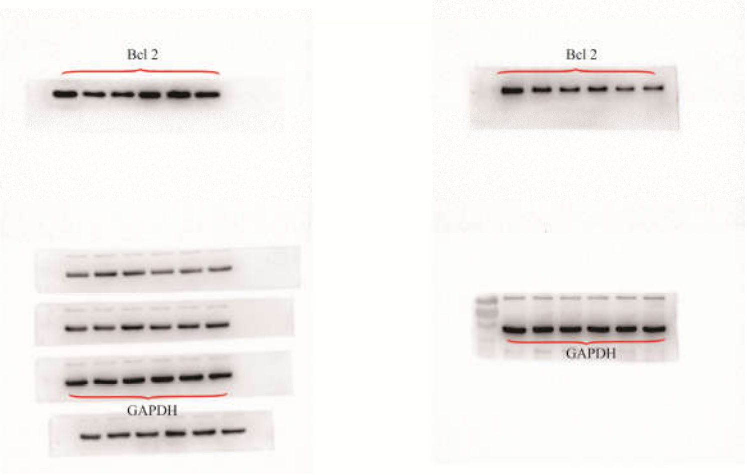

Fig 5.

Original films

a

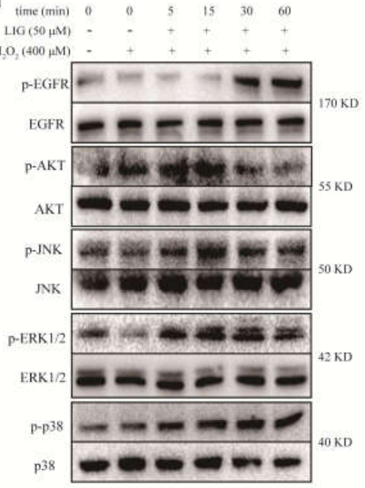

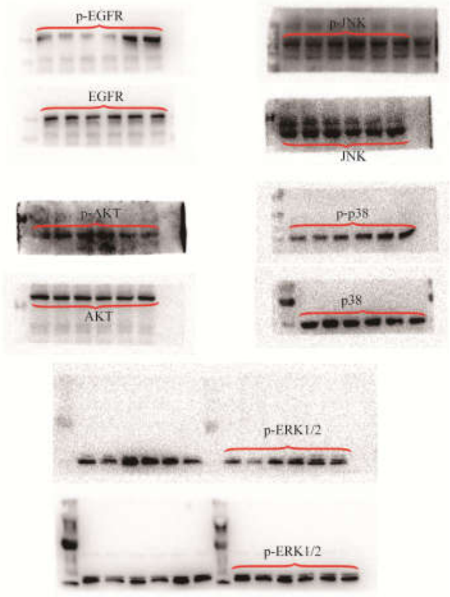

Fig 5.

b

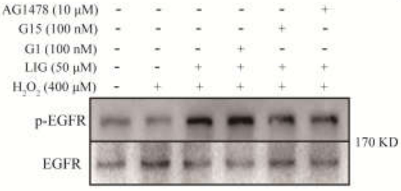

Original films

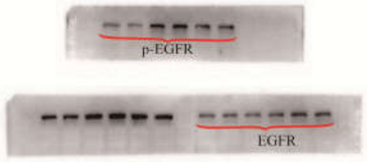

c

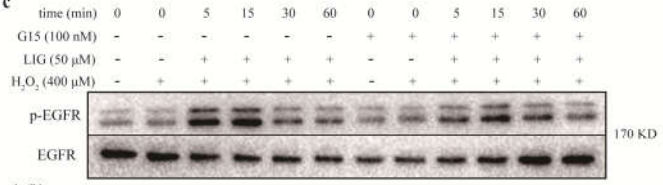

Original films

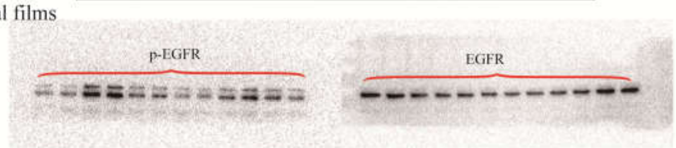

d

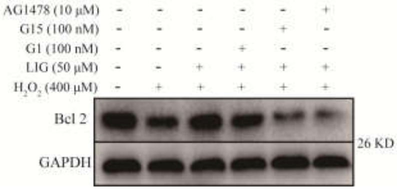

Original films

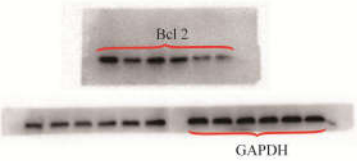

Supplement: Supplementary file 1 — Supplementary Information [file 41598_2019_43518_MOESM1_ESM.pdf]
